# Physical Activity Influences Health-Related Quality of Life in Adults with Juvenile Idiopathic Arthritis

**DOI:** 10.3390/jcm12030771

**Published:** 2023-01-18

**Authors:** Rodrigo Joel de Oliveira, Ana Carolina Londe, Débora Pessoa de Souza, Roberto Marini, Paula Teixeira Fernandes, Simone Appenzeller

**Affiliations:** 1 Graduate Program in Child and Adolescent Health, School of Medical Sciences, University of Campinas (UNICAMP), Campinas 13083-970, Brazil; 2 Department of Pediatrics, School of Medical Sciences, University of Campinas (UNICAMP), Campinas 3083-970, Brazil; 3 Department of Sport Science, Faculty of Physical Education, University of Campinas (UNICAMP), Campinas 13083-851, Brazil; 4 Department of Orthopedics, Rheumatology and Traumatology—School of Medical Sciences, University of Campinas (UNICAMP), Campinas 3083-970, Brazil

**Keywords:** Juvenile Idiopathic Arthritis, sedentary lifestyle, quality of life, physical activity, sport psychology

## Abstract

This cross-sectional study aimed to evaluate the impact of physical activity and physical fitness on the health-related quality of life (HQoL) of adult patients with Juvenile Idiopathic Arthritis (JIA). Fifty-nine JIA patients and sixty healthy individuals participated in this study. All individuals had the following evaluations performed: body composition (electrical bioimpedance), physical fitness (6 min walk test (6MWT)), physical activity level (International Physical Activity Questionnaire (IPAQ)), and HQoL (Quality of Life Questionnaire in relation to Health—Short Form (SF36)). Thirty-nine (66%) JIA patients were considered sedentary compared with 15 (25%) in the control group (*p* < 0.01). JIA patients had a lower HQoL compared with the control group in all variables studied (*p* < 0.05). JIA patients who were very physically active had better HQoL conditions in the categories of functional capacity (*p* = 0.001), limitations by physical aspects (*p* = 0.003), and emotional aspects (*p* = 0.002) compared with sedentary patients. JIA patients had more cardiovascular abnormalities and walked shorter distances compared with healthy controls in the 6MWT. In conclusion, we observed that HQoL was reduced in adults with JIA. A high percentage of JIA patients were sedentary with lower physical fitness, but physically active patients had a better HQoL than sedentary patients. The duration of physical activity, rather than intensity, influenced the mental aspects of HQoL.

## 1. Introduction

Juvenile Idiopathic Arthritis (JIA) is the most common rheumatic inflammatory disease in childhood and comprises a heterogeneous group of diseases characterized by arthritis of unknown origin with an onset before the age of 16 [1]. Although a proportion of patients have a favorable outcome, long-term follow-up has shown that approximately 50% of adults with JIA have persistent active disease and functional impairment (Steinbrocker classes III and IV) [2,3,4,5,6]. The frequency of disability varies according to JIA subtypes and the length of follow-up [2,3,4,5,6]. Despite treatment, functional disability progresses over time, leading to the need for joint replacement in a significant proportion of patients [6,7]. Disability related to extra-articular involvement, such as uveitis (which leads to glaucoma and reduced vision), is also a concern, and its occurrence has been observed in up to 30% of oligoarticular JIA [2,3,4,5,6]. Even in adults with JIA who were functionally independent (Steinbrocker I and II), significant limitations in daily living were observed, especially in mobility and physical activity [8].

Emotional aspects and health-related quality of life (HQoL) have been increasingly studied in JIA patients [6,9,10,11,12,13,14]. Anxiety and depression are comorbidities that have been described to occur in 35.6% and up to 20%, respectively, of adults with JIA, and they were independently associated with a younger age of disease onset, persistent inflammation, perceived body image handicap, and social satisfaction [3].

Although adults with JIA have a better HQoL compared with rheumatoid arthritis (RA) patients, reduced HQoL is observed when they are compared with the general population [10,13]. A reduction in HQoL is associated with pain, functional ability, mood disorders, the burden of medication use, and reduced social activity, including increased school and work absences [11,12,13,14]. JIA patients are also less physically active than their peers and present significantly impaired aerobic and anaerobic exercise capacity, which is more pronounced during disease flares and is associated with pain, mood disorders, and disability [6,15,16,17,18,19,20,21,22,23,24,25,26]. Persistent inflammation, comorbidities, corticosteroid use, increased body fat mass, and reduced muscle mass are associated with an increased risk of cardiovascular disease (CVD) and premature mortality [9,10].

Regular physical activity is a beneficial intervention that prevents and controls several chronic diseases, improves psychological and social health, increases energy expenditure, and reduces premature mortality, especially that arising from CVD [19]. In JIA and RA, physical activity additionally reduces disease activity and radiological disease progression and increases bone mineral density [20,21,22,23,24,25,26]. Adherence to physical activity is generally low but can be improved with low-intensity programs [20].

During the transition period, it is important to ensure that JIA patients can manage their condition independently as adults, addressing not only their medical needs but also their psychosocial, educational, and vocational needs [27]. However, JIA patients without significant pain and disability may be referred to general physicians and not adult rheumatologists, and specific HQoL and emotional aspects may be overlooked in clinical care [28]. Therefore, the objective of our study was to compare the HQoL of adults with JIA without significant disability and pain with that of healthy age- and sex-matched controls and determine the impact of physical activity and physical fitness on HQoL.

## 2. Materials and Methods

### 2.1. Study Project

This cross-sectional study took place between the years 2017 and 2019. The study was conducted in accordance with the Declaration of Helsinki, and this research was approved by the Research Ethics Committee of the State University of Campinas (UNICAMP) (CAAE = 65688017.8.0000.5404).

### 2.2. Participants

We invited consecutive JIA patients aged ≥18 who were followed at the outpatient pediatric rheumatology clinic at the University of Campinas (HC/UNICAMP). JIA was classified according to ILAR criteria [1]. We excluded JIA patients with active disease (Disease Activity Score in 28 Joints (DAS28) ≥ 2.6), patients with pain (visual analog scale (VAS) ≥ 3), and patients with functional impairment (Steinbrocker functional classification > 2) [29]. Fifty-nine JIA patients were included. Sixty healthy individuals with no history of chronic disease matched by sex, age, and sociocultural and economic background were included as controls. The control group consisted of friends and peers of JIA patients, who were invited when accompanying the patients to consults.

### 2.3. Body Composition

Body composition was analyzed using a vertical electrical bioimpedance scale model HBF-514C, brand OMRON^®^ (Bannockburn, Illinois). Initially, the equipment was configured by entering the data of height (meters), age (years), and sex. The subject was positioned on the scale barefoot, where he held a rod that was connected to the equipment at shoulder height. After finishing the reading, which took place in a few seconds, the device read the body weight, body mass index (BMI), percentage of body fat, total muscle mass, and estimated basal metabolic rate.

### 2.4. International Physical Activity Questionnaire (IPAQ)

The International Physical Activity Questionnaire (IPAQ) is a standardized instrument that was created to assess the physical activity level of any individual. It is an open questionnaire that analyzes the practice of physical activity in the last 7 days and can classify the individual into sedentary, irregularly active A and B, active, and very active [30,31]. The following definitions were used:

(a) VERY ACTIVE: One who complies with the recommendations of:
  VIGOROUS: ≥5 days/week and ≥30 min per session;
  VIGOROUS: ≥3 days/week and ≥20 min per session + MODERATE;
  WALKING: ≥5 days/week and ≥30 min per session.
(b) ACTIVE: A person who complies with the recommendations of:
  VIGOROUS: ≥3 days/week and ≥20 min per session;
  MODERATE or WALKING: ≥5 days/week and ≥30 min per session;
  Any activity added together: ≥5 days/week and ≥150 min/week (walking + moderate + vigorous).
(c) IRREGULARLY ACTIVE: One who performs physical activity that is insufficient to be classified as active because it does not meet the recommendations regarding frequency or duration. To receive this classification, the frequency and duration of the different types of activities (walking + moderate + vigorous) are added. This group was divided into two subgroups according to whether some of the recommendation criteria were met:
  (c1)IRREGULARLY ACTIVE A: One who meets at least one of the criteria of the recommendations regarding the frequency or duration of the activity: frequency—5 days/week or duration—150 min/week.
  (c2)IRREGULARLY ACTIVE B: One who does not meet any of the criteria of the recommendations in terms of frequency or duration.
(d) SEDENTARY: One who does not perform any physical activity for at least 10 continuous minutes during the week.

### 2.5. Health-Related Quality of Life Questionnaire—Short Form (SF36)

The Health-Related Quality of Life Questionnaire—Short Form (SF36) is a questionnaire that was developed to assess HQoL and allows the analysis of functional capacity, limitation by physical aspect, pain, general health status, vitality, aspects social, limitations due to emotional aspects, and the mental health of individuals. The SF36 still allows the separation of these mentioned elements, creating two distinct subgroups: a physical and a mental component [32].

### 2.6. Six-Minute Walk Test (6MWT)

The 6 min walk test (6MWT) is widely used to determine the physical fitness of individuals with low physical capacity and those who have some type of physical limitation. It is low-cost and easy to perform [33,34,35]. The participant aims to cover the greatest possible distance in a period of 6 min in the form of a walk. Blood pressure and heart rate values were measured before and after the test. We used the internal corridors of the HC/UNICAMP to carry out the test. The place was covered and ventilated, and two points were marked with a distance of 30 m between them. The performer was monitored for their perceived exertion. The individual could stop the activity at any time.

### 2.7. Statistical Analysis

The statistical analysis of this work was performed using IBM^®^ SPSS Statistics software version 20.0. Fisher’s exact test was used to compare categorical variables. For normally distributed variables, we used Student’s *t*-test. For non-parametric variables, we used the Mann–Whitney *U*-test. SF36 variables and IPAQ scores were compared with *Pearson’s* correlation. The level of significance adopted for this research was *p* < 0.05. Post hoc power analysis was performed using the G*power program [36]. When analyzing the physical and mental components of the SF36 summary scores, we obtained effect sizes (d) of 1.75 and 1.37, respectively.

## 3. Results

We included 59 JIA patients (mean age 29.10 years, standard deviation (SD) = 9.18) and 60 healthy controls (median age = 30.41 ± 3.12 years). The JIA group had a mean disease duration of 18.85 ± 8. 83 years, with mean a DAS 28 score of 2.3 ± 0.98. The most frequently reported JIA subtypes were rheumatoid factor positive polyarticular (37.3%) and rheumatoid factor negative polyarticular (28.8%). Demographic information, disease-related features, and comorbidities are summarized in Table 1.

Pharmacologic treatment was prescribed to 83% of JIA patients. Synthetic DMARDs (sDMARDs) were used by 62.7% of patients, 44% received biological DMARDs (bDMARDs), and 22% of JIA patients used prednisone with a mean dose of 13.85 ± 5.4 mg (Table 1). Only 17% of JIA patients were not taking any DMARDs (Table 1).

Comparing the presence of comorbidities in JIA patients and controls, we observed that JIA patients more frequently had arterial hypertension (12 (20.3%) vs. 5 (8.3%); *p* = 0.05) and diabetes mellitus (8 (13.6%) vs. 2 (3.3%); *p* = 0.045). We did not find significant differences regarding the frequency of dyslipidemia, osteoporosis, or obesity between groups (Table 1).

The control group showed a greater height (169.43 ± 8.11 cm vs. 162.92 ± 9.33 cm; *p* < 0.001), body weight (69.15 ± 6.83 kg vs. 63.56 ± 15.37 kg; *p* = 0.037), and estimated basal metabolism (calories) (1700.25 ± 9.96 vs. 1421.34 ± 23.67; 0.039) compared with JIA patients. BMI, percentage of body fat, and total muscle mass did not differ significantly between groups (Table 2).

Before the start of the 6MWT, JIA patients had significantly higher systolic blood pressure (SBP) (*p* < 0.001) and heart rate (HR) (*p* < 0.001) than controls (*p* < 0.001). The distance covered during the test was lower in the JIA group compared with healthy individuals (498 vs. 569 m; *p*= 0.015). After physical exertion, SBP (*p* = 0.015) and diastolic blood pressure (DBP) (*p* < 0.001) were higher in the JIA group than in the controls. We found no significant difference between the groups in the HR variable after physical exertion (Table 3).

The weekly frequency of walking in the JIA group was lower than that in the control group (0.78 ± 1.43 vs. 2.48 ± 1.80; *p* < 0.001). We also observed a lower time dedicated to walking in JIA patients than in healthy individuals (Table 4). We did not find significant differences between the groups when comparing the weekly frequency of moderate-intensity physical activity (*p* = 0.132); however, the average time devoted to this practice was lower in individuals with JIA (*p* = 0.008). There were no significant statistical differences between the weekly frequency and duration of vigorous-intensity physical activity between the groups in this study (Table 4).

The average time that the groups remained seated during the week was statistically similar. The mean time spent in the sitting position during the weekend was higher in JIA patients (15.14 ± 6.91 vs. 11.08 ± 3.14; *p* < 0.001) (Table 4).

The number of individuals considered sedentary was higher in the JIA group than in the control group (66.1% vs. 25%; *p* < 0.001) (Table 4).

**Figure 1:**
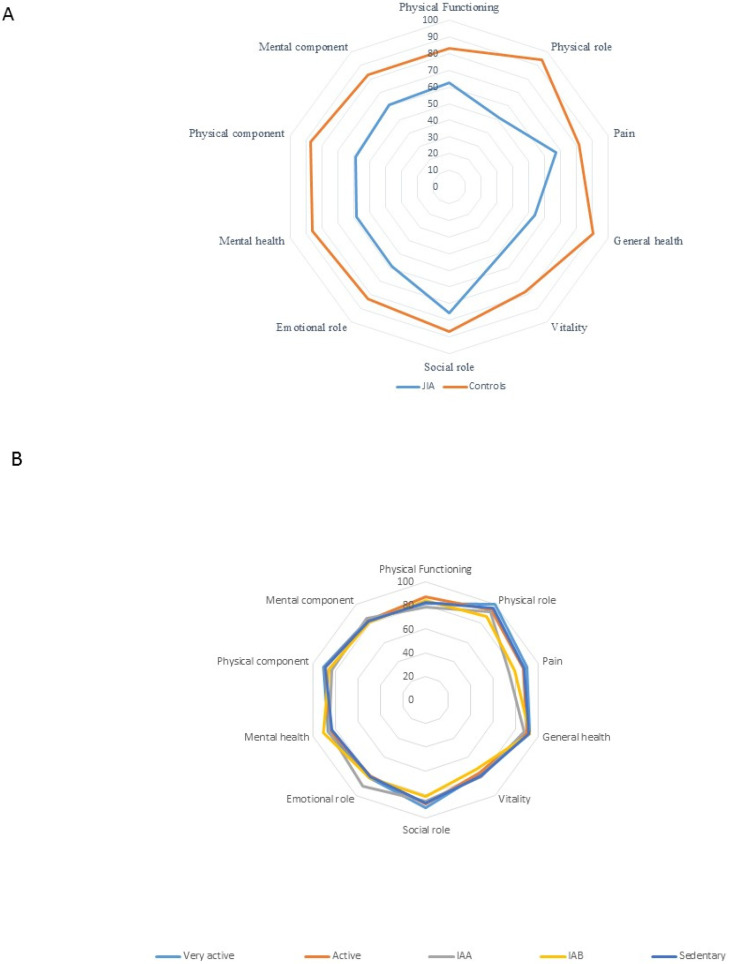

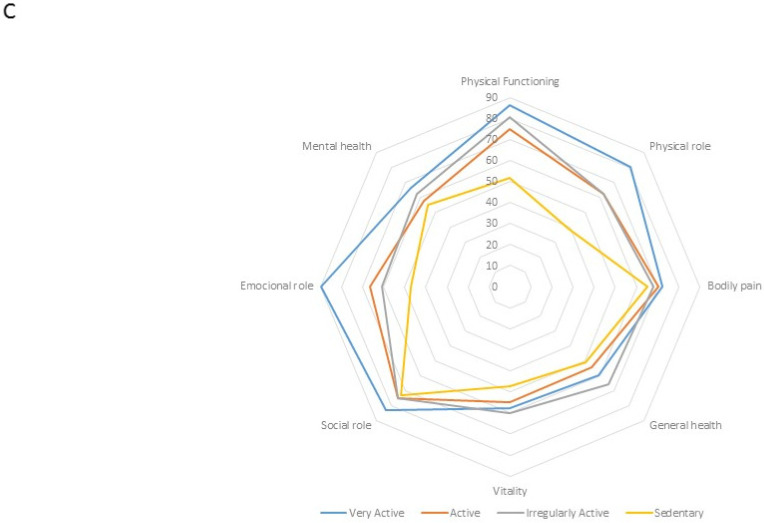
(**A**). Comparison between the SF36 values of JIA patients and the control group. Significant differences were found in all domains (*p* < 0.001 for all analyses except bodily pain (*p* = 0.003) and social role (*p* = 0.015)). (**B**): JIA. Comparison between the mean values of the SF36 variables of each classification element of the physical activity level according to the IPAQ. (**C**): Controls. Comparison between the mean values of the SF36 variables of each classification element of the physical activity level, according to IPAQ.

The HQoL of JIA patients was significantly lower than that of healthy individuals (Figure 1A). JIA patients who were very physically active had better HQoL conditions in the categories of functional capacity (*p* = 0.001), limitations by physical aspects (*p* = 0.003), and emotional aspects (*p* = 0.002) compared with those considered sedentary (Figure 1B). In the control group, physical activity did not influence SF36 scores or subscores (Figure 1C).

Both the frequency (*p* = 0.003; r = 0.071) and the weekly duration (*p* = 0.017; r = 0.029) of vigorous physical activity were positively correlated with the physical component of the SF36 (Table 5).

The mental component of the SF36 was positively correlated with the duration of walking (*p* = 0.036, r = 0.131), moderate-intensity physical activity (*p* = 0.028, r = 0.206), and the frequency (*p* = 0.003, r = 0.193) and duration (*p* = 0.013, r = 0.211) of vigorous physical activity (Table 5).

## 4. Discussion

Long-term follow-up of JIA patients has shown that a significant proportion of patients flare and have progressive disability, emphasizing the need for adult specialist care during the transition period [37,38,39]. HQoL is of concern in these patients since it influences study outcomes, work absenteeism, and social interactions [30,31,32,33,34,35,36,37]. Compared with age- and sex-matched adults, we observed a reduced HQoL in JIA patients, as previously reported [5,10,13,14]. Despite the exclusion from our study of variables previously associated with HQoL in JIA, such as active disease, pain, and physical disability, our cohort still had a worse HQoL compared with controls [5,13,25]. However, in our cohort, only 17% of JIA patients were off medication. The subjective burden of medication use is a known risk factor for worse HQoL in JIA, but we were not able to analyze this effect [5]. We did not observe differences in HQoL between JIA subtypes; however, the small sample size in each group could explain this difference in relation to previously reported results [37].

In our study, we observed that 66% of JIA patients were sedentary. A sedentary lifestyle, characterized by energy expenditure ≤ 1.5 metabolic equivalents (METs), is common in the general population and increases the risk of CVD [40,41]. Approximately 6% of CVD and 7% of type 2 diabetes mellitus (DM2) events are associated with sedentary behavior, directly influencing the financial costs directed toward health in developed countries [38]. In our study, we found that 20% of adult JIA patients had arterial hypertension and 14% had diabetes, a frequency significantly greater than that in healthy controls. During the course of the disease, JIA patients are exposed to inflammatory processes and medications, such as prednisone, that increase the risk of atherosclerosis, CVD, and mortality [42,43,44,45,46,47,48].

In our study, we observed that JIA patients had a lower cardiovascular capacity during the 6MWT test compared with their healthy peers. The 6MWT serves as a monitoring tool in clinical settings, especially for physical activity recommendations, and analyzes patients’ functional capabilities over time [49,50]. We also observed that adult JIA patients walked a significantly shorter distance than healthy controls (498 vs. 570 m, *p* = 0.015), indicating low physical aerobic capacity, as previously reported [51,52]. Improving physical aerobic capacity is associated with CVD risk reduction [53,54]. We also observed that JIA patients spent more time in a sitting position during the weekend compared with controls (15 vs. 11 h; *p* < 0.001), increasingly consolidating the sedentary lifestyle of these individuals. A lack of physical activity reduces the anti-inflammatory response, compromises the cardiorespiratory and musculoskeletal systems, and increases disability [55,56]. Therefore, physical activity can help prevent joint stiffness and deformities, atrophy, and muscle contractures in patients with JIA, enhancing the functional capabilities of these individuals, increasing their longevity, and decreasing the risk of premature mortality [10,11,12,13,14].

In our study, we observed that only 25.4% of adult JIA patients were active or very active. JIA patients who were very physically active had a better HQoL compared with sedentary patients. Previous studies have shown that JIA patients have a low physical capacity, especially in activities of moderate and vigorous intensity [49,57]. The lack of opportunities and stimuli, the severity of the disease and joint pain, and fear are associated with less activity [19,50,58,59]. In addition, low aerobic and anaerobic capacity has been observed in JIA patients with a negative influence on their HQoL [6,16,17,23,50]. The influence of physical activity has mainly been studied in children and adolescents with JIA and has been studied less frequently in adults with JIA [5,11,12,60].

Regular physical activity can positively influence the physical and mental aspects of HQoL [22,49,61,62]. The practice of moderate- or vigorous-intensity physical activity for 75 to 150 min per week increases strength and body flexibility, improving HQoL [63,64,65,66,67,68].

We observed that the physical domains of the SF36 were positively correlated with the duration of moderate activity and the weekly frequency and duration of vigorous activity. The mental component of the SF36 was correlated with walking duration, moderate activity, and the weekly frequency and duration of vigorous activity. These results suggest that to improve the mental aspects of HQoL, the duration of physical activity is more important than the intensity. In a previous study including children with JIA, Pilates was shown to improve both the mental and physical aspects of HQoL [69].

Our study has some limitations. We performed a single-center study and applied strict exclusion criteria in order to exclude pain and disability as risk factors; however, this reduced generalizability. Although we had adequate power, the sample size was small, and we were not able to analyze different JIA subcategories individually. We applied the IPAQ and did not have an objective method to assess physical activity. An intervention study analyzing the effect of an exercise program on HQoL will be our next step.

This study suggests important clinical implications. A high percentage of our adult JIA patients had sedentary behavior, and they had hypertension and diabetes more frequently than the control group. These are relevant CVD risk factors that physicians should address in routine medical consults. Multidisciplinary healthcare team orientation would be of great value to these patients. In addition, we observed that the mental aspect of HQoL may be improved by duration, while the physical aspect was related to the intensity of physical activity.

## 5. Conclusions

In conclusion, sedentary behavior and reduced HQoL were more frequently observed in our adult JIA cohort than in controls. Physical activity was associated with a better HQoL. Thus, the practice of physical activity by these patients should be constantly encouraged by their families and multidisciplinary health teams as an effective part of their treatment plans.

## Figures and Tables

**Table 1:** Distribution of characteristics between groups.

Characteristics	JIA (N = 59)	Controls (N = 60)	*p*
Age in years (mean ± SD)	29.10 ± 9.18	30.41 ± 3.12	0.46
Women N (%)	51 (86.44)	51 (85)	0.82
Disease duration, years (mean ± SD)	18.85 ± 8.83		
DAS 28	2.3 ± 0.98		
Ethnicity			
White (%)	47.5	48.3	
Brown (%)	35.6	31.7	
Black (%)	15.3	15	
Other (%)	1.7	5	
JIA subtypes			
Polyarticular rheumatoid factor negative N (%)	17 (28.8)		
Polyarticular rheumatoid factor positive N (%)	22 (37.3)		
Oligoarticular N (%)	7 (11.9)		
Enthesitis-related arthritis N (%)	5 (8.5)		
Systemic arthritis N (%)	8 (13.6)		
Medications			
Fluoxetine N (%)	1 (0.59)		
Sertraline N (%)	3 (5)		
No DMARD N (%)	10 (17)		
sDMARD N (%)	37 (62.7)		
bDMARD N (%)	26 (44)		
Prednisone N (%)	13 (22)		
Prednisone dose (mg), (mean ± SD)	13.85 ± 5.4		
Comorbidities			
Arterial hypertension	12 (20.3)	5 (8.3)	0.05
Diabetes mellitus	8 (13.6)	2 (3.3)	0.045
Dyslipidemia	10 (16.9)	6 (10)	0.2
Osteoporosis	6 (10.2)	3 (5)	0.237
Obesity	4 (6.8)	3 (5)	0.491

JIA: Juvenile Idiopathic Arthritis; SD: standard deviation; DMARD: disease-modifying antirheumatic drugs; DMARDs: synthetic disease-modifying antirheumatic drugs; DMARDb: biological disease-modifying antirheumatic drugs; DAS28: Disease Activity Score in 28 Joints; mg: milligrams; Fisher’s exact statistical test; significant *p*-value when *p* ≤ 0.05.

**Table 2:** Comparison of means of body composition in JIA patients and controls.

	JIA	Controls	*p*
	(Mean ± SD)	(Mean ± SD)	
Height (cm)	162.92 ± 9.33	169.43 ± 8.11	<0.001
Weight (Kg)	63.56 ± 15.37	69.15 ± 6.83	0.037
BMI (Kg/m ^2^)	24.43 ± 4.74	24.14 ± 2.15	0.915
% Total body fat	31.41 ± 10.25	29.79 ± 6.03	0.583
% Total muscle mass	28.97 ± 7.21	29.81 ± 7.30	0.775
Basal metabolism	1421.34 ± 23.67	1700.25 ± 9.96	0.039

JIA: Juvenile Idiopathic Arthritis; BMI: body mass index; SD: standard deviation.

**Table 3:** Comparison between the means of the 6-minute walk test (6MWT).

	JIA	Controls	*p*
	(Mean ± SD)	(Mean ± SD)	
SBP initial (mmHg)	127.61 ± 8.4	119.07 ± 8.3	<0.001
DBP initial (mmHg)	79.59 ± 6.7	78.27 ± 7.5	0.59
HR (BPM) Initial	90.68 ± 15.3	77.18 ± 13.14	<0.001
Distance covered, meters	498.05 ± 14.6	569.78 ± 13.0	0.015
SBP final (mmHg)	154.8 ± 7.6	150.68 ± 8.9	0.015
DBP final (mmHg)	86.19 ± 5.9	80.25 ± 7.0	<0.001
HR (BPM) final	140.63 ± 15.0	138.87 ± 9.0	0.744

JIA: Juvenile Idiopathic Arthritis; SBP: systolic blood pressure; DBP: diastolic blood pressure; HR (BPM): heart rate (beats per minute); SD: standard deviation.

**Table 4:** Comparison between the means of the International Physical Activity Questionnaire (IPAQ).

	JIA	Controls	*p*
Walking frequency	0.78 ± 1.43	2.48 ± 1.80	<0.001
Walking duration	6.86 ± 12.76	18.50 ± 14.62	<0.001
Frequency of moderate physical activity	0.44 ± 1.03	0.83 ± 1.16	0.132
Duration of moderate physical activity	4.92 ± 11.04	11.86 ± 13.96	0.008
Frequency of vigorous physical activity	0.90 ± 1.70	0.82 ± 1.50	0.96
Duration of vigorous physical activity	5.93 ± 11.00	7.17 ± 12.22	0.833
Time sitting during the week	35.36 ± 14.92	31.33 ± 7.12	0.134
Time sitting during the weekend	15.14 ± 6.91	11.08 ± 3.14	<0.001
Very active N (%)	13 (22)	9 (15)	0.226
Active N (%)	2 (3.4)	13 (21.7)	0.002
IAA N (%)	2 (3.4)	14 (23.3)	0.001
IAB N (%)	3 (5.1)	9 (15)	0.067
Sedentary N (%)	39 (66.1)	15 (25)	<0.001

JIA: Juvenile Idiopathic Arthritis; IAA: irregularly active A; IAB: irregularly active B; SD: standard deviation.

**Table 5:** Correlation between the mean scores of the IPAQ with the scores of the physical and mental components of the Health-Related Quality of Life Assessment Questionnaire (SF36) of patients with JIA and the control group.

	JIA		Controls	
Physical Component	r	*p*	r	*p*
Frequency of walking	−0.066	0.022	−0.093	0.583
Walking duration	−0.054	0.009	−0.115	0.489
Frequency of moderate physical activity	−0.001	0.116	0.138	0.683
Duration of moderate physical activity	0.027	0.014	0.116	0.655
Frequency of vigorous physical activity	0.071	0.003	0.212	0.989
Duration of vigorous physical activity	0.029	0.017	0.161	0.927
Sitting time during the week	−0.31	0.173	0.001	0.25
Sitting time during the weekend	−0.341	0.403	−0.138	0.496
Mental component				
Frequency of walking	0.14	0.072	−0.153	0.643
Walking duration	0.131	0.036	−0.099	0.655
Frequency of moderate physical activity	0.207	0.186	0.111	0.462
Duration of moderate physical activity	0.206	0.028	−0.063	0.481
Frequency of vigorous physical activity	0.193	0.003	0.308	0.939
Duration of vigorous physical activity	0.211	0.013	0.301	0.859
Sitting time during the week	−0.184	0.373	−0.14	0.964
Sitting time during the weekend	−0.288	0.541	0.001	0.859

r: Pearson’s Correlation Coefficient; Significant *p*-value when *p* ≤ 0.05.

## Data Availability

The raw data supporting the conclusions of this article will be made available by the authors without undue reservation.

## References

[1] Petty R.E., Southwood T.R., Manners P., Baum J., Glass D.N., Goldenberg J., He X., Maldonado-Cocco J., Orozco-Alcala J., Prieur A.-M. (2004). International League of Associations for Rheumatology classification of juvenile idiopathic arthritis: Second revision, Edmonton, 2001. J. Rheumatol..

[2] Fantini F., Gerloni V., Gattinara M., Cimaz R., Arnoldi C., Lupi E. (2003). Remission in juvenile chronic arthritis: A cohort study of 683 consecutive cases with a mean 10-year follow-up. J. Rheumatol..

[3] Packham J.C., Hall M.A. (2002). Long-term follow-up of 246 adults with juvenile idiopathic arthritis: Functional outcome. Rheumatology.

[4] Bertilsson L., Andersson-Gare B., Fasth A., Petersson I.F., Forsblad-D’elia H. (2003). Disease course, outcome, and predictors of outcome in a population-based juvenile chronic arthritis cohort followed for 17 years. J Rheumatol..

[5] Tollisen A., Selvaag A.M., Aulie H.A., Lilleby V., Aasland A., Lerdal A., Flatø B. (2018). Physical Functioning, Pain, and Health-Related Quality of Life in Adults with Juvenile Idiopathic Arthritis: A Longitudinal 30-Year Follow up Study. Arthritis Care Res..

[6] Van Pelt P.A., Takken T., van Brussel M., de Witte M., Kruize A.A., Wulffraat N.M. (2012). Aerobic capacity and disease activity in children, adolescents and young adults with juvenile idiopathic arthritis (JIA). Pediatr. Rheumatol. Online J..

[7] Laaksonen A. (1966). A prognostic study of juvenile rheumatoid arthritis: Analysis of 544 cases. Acta Paediatr. Scand..

[8] Doherty E., Rooney M., Coneroy R., Bresnithan B. (1988). Health status of functionally independent young adults with chronic arthritis since childhood. J. Orthop. Rheumatol..

[9] Gabriel S.E., Michaud K. (2009). Epidemiological studies in incidence, prevalence, mortality, and comorbidity of the rheumatic diseases. Arthritis Res. Ther..

[10] French A.R., Mason T., Nelson A.M., O’Fallon W.M., Gabriel S.E. (2001). Increased mortality in adults with a history of juvenile rheumatoid arthritis: A population-based study. Arthritis Rheum..

[11] Haverman L., Grootenhuis M.A., van den Berg J.M., van Veenendaal M., Dolman K.M., Swart J.F., Kuijpers T.W., van Rossum M.A. (2012). Predictors of health-related quality of life in children and adolescents with juvenile idiopathic arthritis: Results from a web-based survey. Arthritis Care Res..

[12] Rebane K., Ristolainen L., Relas H., Orenius T., Kautiainen H., Luosujärvi R., Aalto K., Säilä H. (2019). Disability and health-related quality of life are associated with restricted social participation in young adults with juvenile idiopathic arthritis. Scand. J. Rheumatol..

[13] Barth S., Haas J.P., Schlichtiger J., Molz J., Bisdorff B., Michels H., Hügle B., Radon K. (2016). Long-term health-related quality of life in German patients with juvenile idiopathic arthritis in comparison to the German general population. PLoS ONE.

[14] Tollisen A., Selvaag A.M., Aasland A., Ingebrigtsen T., Sagen J., Lerdal A., Flatø B. (2022). Personally-Generated Quality of Life Outcomes in Adults with Juvenile Idiopathic Arthritis. J. Rheumatol..

[15] Nesbitt C., Kuntze G., Toomey C., Esau S., Brooks J., Mosher D., Emery C.A. (2022). Secondary consequences of juvenile idiopathic arthritis in children and adolescents with knee involvement: Physical activity, adiposity, fitness, and functional performance. Rheumatol. Int..

[16] Hulsegge G., Henschke D., McKay Chaitow J., West K., Broderick C., Singh-Grewal D. (2015). Fundamental movement skills, physical fitness and physical activity among Australian children with juvenile idiopathic arthritis. J. Paediatr. Child Health.

[17] Doğrupti M., Kasapcopur O., Mengi M., Öztürk G., Metin G. (2014). Regular aerobic training combined with range of motion exercises in juvenile idiopathic arthritis. BioMed Res. Int..

[18] Klepper S.E. (2003). Exercise and fitness in children with arthritis: Evidence of benefits for exercise and physical activity. Arthritis Rheum..

[19] Warburton D.E., Nicol C.W., Bredin S.S. (2006). Health benefits of physical activity: The evidence. CMAJ.

[20] Takken T., Van Brussel M., Engelbert R.H., Van Der Net J., Kuis W., Helders P.J. (2008). Exercise therapy in juvenile idiopathic arthritis: A Cochrane Review. Eur. J. Phys. Rehabil. Med..

[21] Long A.R., Rouster-Stevens K.A. (2010). The role of exercise therapy in the management of juvenile idiopathic arthritis. Curr. Opin. Rheumatol..

[22] Carandang K., Vigen C.L.P., Ortiz E., Pyatak E.A. (2019). Re-conceptualizing functional status through experiences of young adults with inflammatory arthritis. Rheumatol. Int..

[23] Perandini L.A., de Sá-Pinto A.L., Roschel H., Benatti F.B., Lima F.R., Bonfá E., Gualano B. (2012). Exercise as a therapeutic tool to counteract inflammation and clinical symptoms in autoimmune rheumatic disease. Autoimmun. Rev..

[24] Fransen J., Creemers M.C., Van Riel P.L. (2004). Remission in rheumatoid arthritis: Agreement of the disease activity score (DAS28) with the ARA preliminary remission criteria. Rheumatology.

[25] Iversen M.D., Andre M., von Heideken J. (2022). Physical Activity Interventions in Children with Juvenile Idiopathic Arthritis: A Systematic Review of Randomized Controlled Trials. Pediatr. Health Med Ther..

[26] Oliveira Ramos F., Rodrigues A., Magalhaes Martins F., Melo A.T., Aguiar F., Brites L., Azevedo S., Duarte A.C., Furtado C., Mourão A.F. (2021). Health-related quality of life and disability in adults with juvenile idiopathic arthritis: Comparison with adult-onset rheumatic diseases. RMD Open.

[27] Ramos F.O., Rodrigues A., Martins F.M., Melo A.T., Aguiar F., Brites L., Azevedo S., Duarte A.C., Furtado C., Mourão A.F. (2017). EULAR/PReS standards and recommendations for the transitional care of young people with juvenile-onset rheumatic diseases. Ann. Rheum. Dis..

[28] Costello R.E., Kearsley-Fleet L., McDonagh J.E., Hyrich K.L., Humphreys J.H. (2022). Continuing specialist care into adulthood in young people with juvenile idiopathic arthritis: A retrospective cohort study using electronic health records in England. Rheumatology.

[29] Steinbrocker O., Traeger C.H., Batterman R.C. (1949). Therapeutic criteria in rheumatoid arthritis. JAMA.

[30] Wolin K.Y., Heil D.P., Askew S., Matthews C.E., Bennett G.G. (2008). Validation of the International Physical Activity Questionnaire-Short among Blacks. J. Phys. Act. Health.

[31] Matsudo S., Araújo T., Matsudo V., Andrade D., Andrade E., Oliveira L.C., Braggion G. (2001). Questionário Internacional de Atividade Física (IPAQ): Estudo de validade e reprodutibilidade no Brasil. Ativ. Física Saúde.

[32] Ciconelli R.M., Ferraz M.B., Santos W., Meinão I., Quaresma M.R. (1999). Tradução para a língua portuguesa e validação do questionário genérico de avaliação de qualidade de vida SF-36 (Brasil SF-36). Rev. Bras. Reumatol..

[33] ATS-AMERICAN THORACIC SOCYET (2002). ATS Statement: Guidelines For The Six-Minute Walk Test. Am. J. Respir. Care Med..

[34] Schveitzer V., Claudino R., Ternes M. (2009). Teste de caminhada de seis minutos: Passos para realizá-lo. Rev. Dig..

[35] Rezende R.R., Nogueira OADal Corso S., Malaguti C. (2009). Uma atualização e Proposta de Padronização do Teste De Caminhada Dos Seis Minutos. Rev. Fisioter. Mov..

[36] Faul F., Erdfelder E., Lang A.-G., Buchner A. (2007). G*Power 3: A flexible statistical power analysis program for the social, behavioral, and biomedical sciences. Behav. Res. Methods.

[37] Mikola K., Rebane K., Arnstad E.D., Berntson L., Fasth A., Glerup M., Herlin T., Kautiainen H., Nielsen S., Nordal E. (2022). Transitioning patients with juvenile idiopathic arthritis to adult care: The Nordic experience. Pediatr. Rheumatol. Online J..

[38] Smitherman E.A., Chahine R.A., Bitencourt N., Rahman A.F., Lawson E.F., Chang J.C. (2023). CARRA Registry Investigators and CARRA Transition Workgroup. Patient-Reported Outcomes among Transition-Age Young Adults with Juvenile Idiopathic Arthritis in the Childhood Arthritis and Rheumatology Research Alliance Registry. J. Rheumatol..

[39] Rebane K., Orenius T., Ristolainen L., Relas H., Kautiainen H., Luosujärvi R., Säilä H., Aalto K. (2019). Pain interference and associated factors in young adults with juvenile idiopathic arthritis. Scand. J. Rheumatol..

[40] Dempsey P.C., Biddle S.J.H., Buman M.P., Chastin S., Ekelund U., Friedenreich C.M., Katzmarzyk P.T., Leitzmann M.F., Stamatakis E., van der Ploeg H.P. (2020). New global guidelines on sedentary behaviour and health for adults: Broadening the behavioural targets. Int. J. Behav. Nutr. Phys. Act..

[41] Santana C.P., Nunes H.A.S., Silva A.N., Azeredo C.M. (2021). Associação entre supervisão parental e comportamento sedentário e de inatividade física em adolescentes brasileiros [Association between parental supervision and sedentary behavior and physical inactivity and among Brazilian adolescents]. Cien. Saude Colet..

[42] Klepper S.E. (2008). Exercise in pediatric rheumatic diseases. Curr. Opin. Rheumatol..

[43] Cavallo S., April K.T., Grandpierre V., Majnemer A., Feldman D.E. (2014). Leisure in children and adolescents with juvenile idiopathic arthritis: A systematic review. PLoS ONE.

[44] Gualano B., Sá Pinto A.L., Perondi B., Leite Prado D.M., Omori C., Almeida R.T., Sallum A.M., Silva C.A. (2010). Evidence for prescribing exercise as treatment in pediatric rheumatic diseases. Autoimmun. Rev..

[45] Van der Net J., van der Torre P., Engelbert R.H., Engelen V., van Zon F., Takken T., Helders P.J. (2008). Motor performance and functional ability in preschool- and early school-aged children with Juvenile Idiopathic Arthritis: A cross-sectional study. Pediatr. Rheumatol. Online J..

[46] Bueno D.R., Marucci Mde F., Codogno J.S., de Roediger M.A. (2016). Os custos da inatividade física no mundo: Estudo de revisão [The costs of physical inactivity in the world: A general review]. Cien. Saude Colet..

[47] Coulson E.J., Ng W.F., Goff I., Foster H.E. (2013). Cardiovascular risk in juvenile idiopathic arthritis. Rheumatology.

[48] Feldman D.E., Vinet É., Bérard A., Duffy C., Hazel B., Meshefedjian G., Sylvestre M.P., Bernatsky S. (2017). Heart Disease, Hypertension, Gestational Diabetes Mellitus, and Preeclampsia/Eclampsia in Mothers With Juvenile Arthritis: A Nested Case-Control Study. Arthritis Care Res..

[49] Tian Z., Mclaughlin J., Verma A., Chinoy H., Heald A.H. (2021). The relationship between rheumatoid arthritis and diabetes mellitus: A systematic review and meta-analysis. Cardiovasc. Endocrinol. Metab..

[50] Suppasit P., Vilaiyuk S., Poomthavorn P., Pongratanakul S., Khlairit P., Mahachoklertwattana P. (2022). Glucose metabolism in systemic juvenile idiopathic arthritis. Pediatr. Rheumatol. Online J..

[51] Lee H., Jin Y., Liu J., Cohen E.M., Chen S.K., Kim S.C. (2020). Risk of Diabetes Mellitus in Patients with Juvenile Idiopathic Arthritis. J. Rheumatol..

[52] Woolnough L.U., Lentini L., Sharififar S., Chen C., Vincent H.K. (2022). The relationships of kinesiophobia and physical function and physical activity level in juvenile idiopathic arthritis. Pediatr. Rheumatol. Online J..

[53] Pritchard L., Verschuren O., Roy M., Kaup C., Rumsey D.G. (2022). Reproducibility of the Six-Minute Walk Test in Children and Youth with Juvenile Idiopathic Arthritis. Arthritis Care Res..

[54] Mian Q., Rumsey D.G., Verschuren O., Moez E.K., Roy M., Kaup C., Pritchard L. (2022). Reference Values for the Six Minute Walk Test in Children with Juvenile Idiopathic Arthritis. Phys. Occup. Ther. Pediatr..

[55] Lee D.C., Artero E.G., Sui X., Blair S.N. (2010). Mortality trends in the general population: The importance of cardiorespiratory fitness. J. Psychopharmacol..

[56] Melo X., Santa-Clara H., Santos D.A., Pimenta N.M., Minderico C.S., Fernhall B., Sardinha L.B. (2015). Linking cardiorespiratory fitness classification criteria to early subclinical atherosclerosis in children. Appl. Physiol. Nutr. Metab..

[57] Pi H., Zhou H., Jin H., Ning Y., Wang Y. (2017). Abnormal Glucose Metabolism in Rheumatoid Arthritis. Biomed Res. Int..

[58] Bilski J., Brzozowski B., Mazur-Bialy A., Sliwowski Z., Brzozowski T. (2014). The role of physical exercise in inflammatory bowel disease. Biomed Res. Int..

[59] Rochette E., Duché P., Merlin E. (2015). Juvenile idiopathic arthritis and physical activity: Possible inflammatory and immune modulation and tracks for interventions in young populations. Autoimmun. Rev..

[60] Bohr A.H., Nielsen S., Müller K., Karup Pedersen F., Andersen L.B. (2015). Reduced physical activity in children and adolescents with Juvenile Idiopathic Arthritis despite satisfactory control of inflammation. Pediatr. Rheumatol. Online J..

[61] Bartlett D.B., Willis L.H., Slentz C.A., Hoselton A., Kelly L., Huebner J.L., Kraus V.B., Moss J., Muehlbauer M.J., Spielmann G. (2018). Ten weeks of high-intensity interval walk training is associated with reduced disease activity and improved innate immune function in older adults with rheumatoid arthritis: A pilot study. Arthritis Res. Ther..

[62] Baillet A., Vaillant M., Guinot M., Juvin R., Gaudin P. (2012). Efficacy of resistance exercises in rheumatoid arthritis: Meta-analysis of randomized controlled trials. Rheumatology.

[63] Martini A., Lovell D.J., Albani S., Brunner H.I., Hyrich K.L., Thompson S.D., Ruperto N. (2022). Juvenile idiopathic arthritis. Nat. Rev. Dis. Prim..

[64] US Centers for Disease Control and Prevention Physical Activity and Health: A Report of the Surgeon General Executive Summary. https://www.cdc.gov/nccdphp/sgr/.

[65] Sandstedt E., Fasth A., Eek M.N., Beckung E. (2013). Muscle strength, physical fitness and well-being in children and adolescents with juvenile idiopathic arthritis and the effect of an exercise programme: A randomized controlled trial. Pediatr. Rheumatol. Online J..

[66] Van Oort C., Tupper S.M., Rosenberg A.M., Farthing J.P., Baxter-Jones A.D. (2013). Safety and feasibility of a home-based six week resistance training program in juvenile idiopathic arthritis. Pediatr. Rheumatol. Online J..

[67] Elhai M., Bazeli R., Freire V., Feydy A., Drapé J.L., Quartier P., Kahan A., Deslandre C., Wipff J. (2013). Radiological peripheral involvement in a cohort of patients with polyarticular juvenile idiopathic arthritis at adulthood. J. Rheumatol..

[68] Perrin J.M., Van Cleave J., Anderson L.E. (2014). The rise in chronic conditions among infants, children, and youth can be met with continued health system innovations. J. Health Aff..

[69] Azab A.R., Kamel F.H., Basha M.A., Alrawaili S.M., Aloraini G.S., Hassan S.M., Ewais N.F., Elnaggar R.K. (2022). Impact of Clinical Pilates Exercise on Pain, Cardiorespiratory Fitness, Functional Ability, and Quality of Life in Children with Polyarticular Juvenile Idiopathic Arthritis. Int. J. Environ. Res. Public Health.

